# Case of Compound Fracture of the Skull, Followed by Hernia Cerebri; Death Thirty-Two Days after the Injury

**Published:** 1838-12-05

**Authors:** Henry H. Smith

**Affiliations:** Resident Surgeon


*Case of Compound Fracture of the Skull, followed by Hernia Cerebri; death thirty-two days after the injury.*

Wm. H——, æt. thirty-five years, was admitted on the evening of the 22d of October, 1838, for a compound fracture of the skull, caused by the kick of a horse four hours before his entrance. On his admission there was found a wound three inches long in the scalp, and a comminuted fracture of the skull, for the same distance, near the junction of the occipital bone, with the parietal of the right side. Several pieces of the cranium had been removed previous to admission, and several more afterwards. A slight superficial wound, also, existed over the right ear; pulse slow, but feeble; answers slowly, not altogether rationally; pupils natural; no paralysis; head shaved, cold applied to it, and sinapisms to extremities.

23*d.*—Rested well; pulse quick and febrile; skin hot and dry; head hot; perfectly sensible; complains of no pain; ordered V. S. to ℥viii.; R. cal. jalap. āā gr. x.; poultice to wound.

25*th.*—Pulse risen since last bleeding; fever continues; otherwise doing well. R. mist. neut. ℥vi., ant. tart. gr. ss., ℥ss. q. 2dâ h.

27*th.*—Sleeps well; tongue natural; bowels torpid; pulse slow and regular, eighty in the minute; no fever; wound suppurating a little on the edges; perfectly rational, but inclined to dose. Ordered blister to the neck, and cal. gr. one-fourth q. 2dâ h., until it touches the gums.

The blister drew well and was kept open for several days; his gums became slightly sore, and he remained without a bad symptom from October 28th till November 8th, during which time the wound was poulticed, and his diet kept reduced.

9*th.*—Wound suppurating freely, but shows no disposition to granulate. Ordered tinct. valer. fℨj. q. ter. horâ, and broth and vegetables for diet.

11*th.*—Wound has the same appearance; bone bare for an inch at the extremity of the wound; dura mater becoming prominent; pulsation of brain perfectly distinct.

13*th.*—Wound sloughing; dura mater ruptured last night; considerable discharge in the dressings; brain beginning to protrude.

14*th.*—Cerebral mass has risen on a level with outside of the cranium; pulsations distinct in it; patient perfectly rational; complains of no pain; gums still sore. Ordered low diet, and compresses of lint, wet with lime water, to the protruding matter.

19*th.*—Since last date the tumour has gone on gradually increasing, is now above the scalp, and of the size of a large hen's egg; patient comprehends questions readily, but is slow in enunciating; pulse regular, but feeble, about eighty-seven in the minute; pupils natural; pressure on the tumor causes them to dilate for the instant, but they soon resume their natural size; continue pressure, and low diet; mist. neut. and ant. tart. continued.

21*st.*—Tumour sloughing; cerebral matter mixed with pus, and some blood, constantly discharging; pulse one hundred and thirty, quick and febrile; skin hot; patient restless, complains of no pain; intellect not clear, but is still rational.

22*d.*—Pulse very quick and feeble; has been delirious since ten o'clock last night; pupils dilated; some diarrhœa; water drawn off by catheter. Omit mist. neut., and allowed any diet he wishes; ordered laud. gtt. xxx., by injection; poultice to wound.

23*d.*—Died at three A.M., twenty-nine hours after he became delirious. No perfect examination of the head could be obtained. But permission being given, the tumour was cut off. This was followed by a free discharge of pure pus and sanious matter from within the brain. A large abscess, near three inches deep, was now evident in the substance of the brain, immediately under the tumour. A second fracture, with depression of the bone, was also found over the ear, where the second wound, spoken of before, was seen. The abscess extended as far forward and outwards as this point, and was apparently lined by a false membrane.

This case has been considered of more than ordinary interest, from its being the second which has occurred in the hospital during the last six months, both of which have presented nearly the same appearances. and tend to establish the point of the hernia being caused by the formation of the abscess below. In the first case, (reported by Dr. Meigs, in number eighteen,) "it was evident that the tumour consisted of the substance of the brain; also, that an abscess had existed near the base of the tumour, and that there was no blood effused, either beneath the bone or dura mater, nor was there any apoplectic cavity." In the second case, although a complete examination could not be made, yet enough was seen to satisfy those present that the tumour consisted of cerebral matter,—that there was no proper fungus,—and that a large abscess, secreting pure pus, had existed under the tumour. This abscess was probably caused by the irritation of the depressed bone over the ear, though no positive proof of it could be given. Yet the situation, and the length of time during which the patient remained without any bad symptoms, might lead us to suppose so. As far, therefore, as these cases would enable us to judge, there can be little doubt as to the fact of the hernia cerebri being properly a protrusion of the cerebral substance, caused by the formation of extraneous matter within the brain, and that there is, in such cases, nothing of a fungus growth connected with it, as has been asserted by some writers. Should this view of the formation of the tumour be admitted, there can be little doubt as to the treatment. For an incision in the dura mater, as soon as fluctuation was evident, would enable us to prevent the protrusion of the brain, and, by evacuating the fluid, allow the sides of the abscess to come together; while the severe laceration, which the brain has suffered without any material injury to the patient, would afford us good reason to believe that a cure might be effected by an operation, which has been so long proposed but so seldom practised.

